# ﻿*Fordiophytontereticaule* (Melastomataceae), a new species from China

**DOI:** 10.3897/phytokeys.197.82670

**Published:** 2022-05-27

**Authors:** Si-Jin Zeng, Yi-Hua Tong, Nian-He Xia

**Affiliations:** 1 Key Laboratory of Plant Resources Conservation and Sustainable Utilization, South China Botanical Garden, Chinese Academy of Sciences, Guangzhou 510650, China South China Botanical Garden, Chinese Academy of Sciences Guangzhou China; 2 Center of Conservation Biology, Core Botanical Gardens, Chinese Academy of Sciences, Guangzhou 510650, China Core Botanical Gardens, Chinese Academy of Sciences Guangzhou China

**Keywords:** phylogeny, Sonerileae, taxonomy, Yunnan

## Abstract

A new species, *Fordiophytontereticaule*, from China, is described and illustrated here based on morphological and molecular evidence. It is morphologically similar to *F.faberi* in having erect stems, slightly oblique and membranous leaf blades, broadly ovate to suborbicular bracts, and oblong petals, but differs by the terete stems, densely puberulous petioles, and elliptic leaf blades. Our phylogenetic analyses based on plastid genome and nrITS data indicate that this new species is clustered with four *Fordiophyton* species of Yunnan but placed far apart from *F.faberi*. An updated key to the genus is also provided.

## ﻿Introduction

*Fordiophyton* Stapf, a genus belonging to the tribe Sonerileae of the family Melastomataceae, is endemic to China and Vietnam with 15 species currently known (Chen 1984; Chen and Renner 2007; Ning and Liu 2010; Zeng et al. 2016a, b; Dai et al. 2019; Dai et al. 2020). All species occur in South China except *F.phamhoangii* (V.T.Pham, C.T.Vu & Ranil) T.V.Do & Ying Liu which is endemic to Vietnam. *Fordiophytonfaberi* Stapf was designated as the type of *Fordiophyton* (Deng and Wu 2004). This genus is characterized as usually having raphides, 4-merous flowers and eight distinctly dimorphic and unequal stamens without ventral tubercles or a dorsal spur at the connective base (Deng and Wu 2004; Chen and Renner 2007; Zeng et al. 2016a). Recent phylogenetic studies have shown that *Fordiophyton* is close to *Blastus* Lour. and *Bredia* Blume (Zeng et al. 2016a; Zhou et al. 2019a, b).

During a recent survey in Malipo County, Yunnan Province, China in November 2019, we encountered an interesting *Fordiophyton* species with terete stems. At first glance, it looked like another two species also occurring in Yunnan, viz. *F.faberi* Stapf or *F.strictum* Diels because of its erect stems and membranous leaf blades, but those two species have very different quadrangular stems.

After careful examination of specimens of *Fordiophyton* species from China and Vietnam, and referring to the relevant references (Chen 1984; Pham 2000; Chen and Renner 2007; Ning and Liu 2010; Zeng et al. 2016a, b; Dai et al. 2019; Dai et al. 2020), we were unable to match this unknown species with any previously recorded species. We thus describe it here as a new species. To evaluate its phylogenetic position and relationships with congeneric species, phylogenetic analysis based on plastid genome and nrITS data was performed.

## ﻿Materials and methods

A total of 43 species from 13 genera (including 14 species of *Fordiophyton*) in the Sonerileae were sampled (Suppl. material 1: Table S1). *Sarcopyramisnapalensis* Wall. and *Sonerilacantonensis* Stapf were selected as outgroup taxa according to Zhou et al. (2019a). All the plastid genome and nrITS sequences were downloaded from Genbank except that of this unknown species.

Silica-gel dried leaves of this unknown species were sent to Novogene (Tianjin, China) to extract total genomic DNA for library (350 bp) preparation for genome skimming sequencing. Paired-end (150 bp) sequencing was conducted on Illumina NovaSeq 6000 (San Diego, CA, USA), generating ca. 20 Gb raw data. After quality control of the raw data by fastp v.0.23 (Chen et al. 2018), ca. 6 Gb paired reads were extracted for the plastid and rDNA assembly by GetOrganelle v.1.7 (Jin et al. 2020), and the plastid genome (MK994846) of *F.faberi* and the rDNA (KM117261) of *Wisteriafloribunda* (Willd.) DC. were used as the reference. Plastid Genome Annotator (Qu et al. 2019) and Geneious Prime 2019 (<www.geneious.com>) were used for the annotation of the plastid genome. The nrITS sequence of this sample was extracted from the rDNA by Geneious.

All plastid genome sequences were aligned by MAFFT v.7.4 (FFT-NS-i × 1000 strategy) after removing one inverted repeat region of each sample (Katoh and Standley 2013). The nrITS data were aligned in MEGA v.7 using MUSCLE (Edgar 2004; Kumar et al. 2016). Maximum likelihood analyses were conducted by IQTREE v.1.6 using SH-aLRT test and ultrafast bootstrap (UFBoot) feature (–alrt 1000 –bb 1000) on CentOS v.7.6 (Nguyen et al. 2015; Hoang et al. 2018).

## ﻿Results of phylogenetic analyses

**Figure 1.**
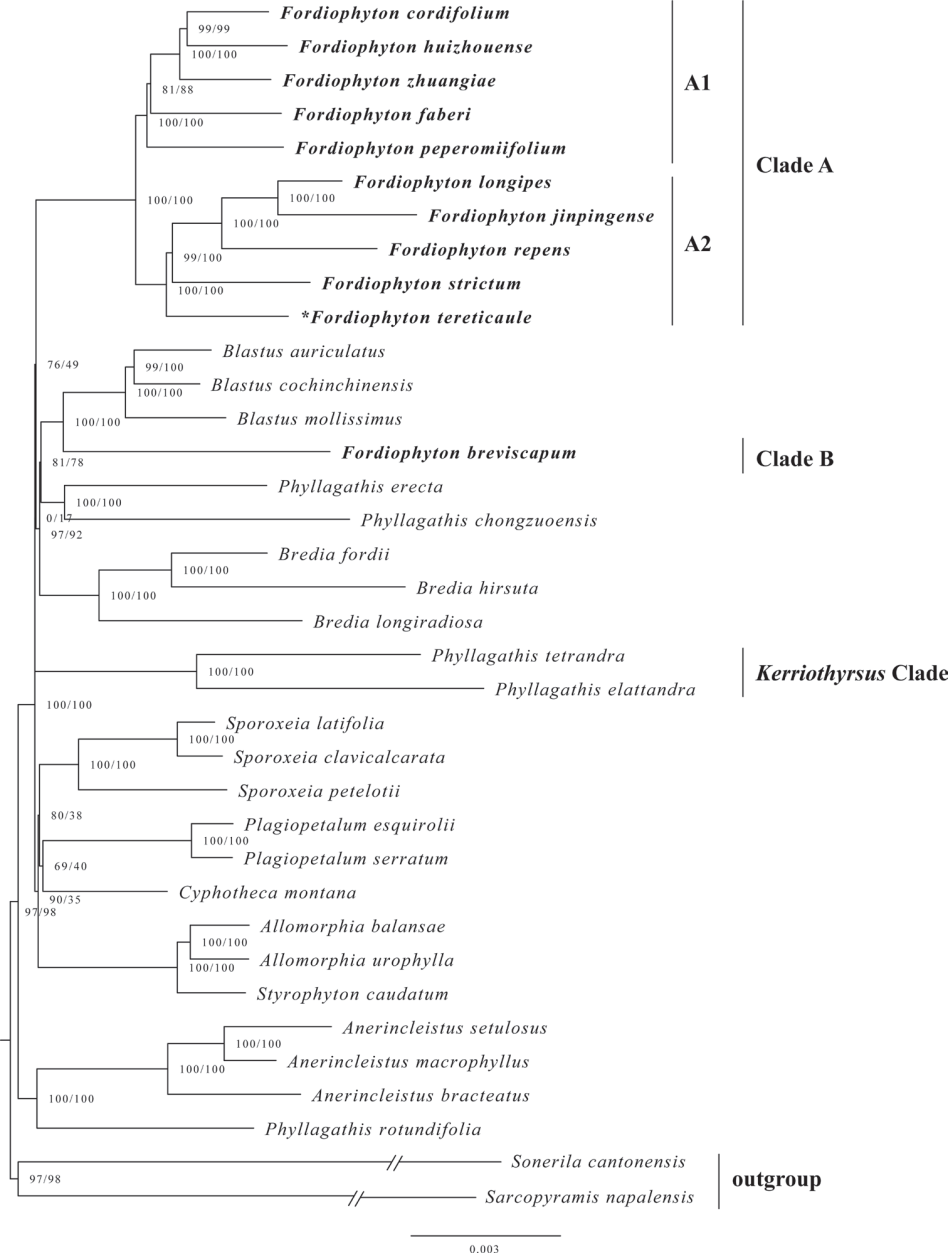
Phylogenetic relationships of Sonerileae based on plastid genome. The numbers near the nodes are the values of the SH-aLRT test (left) and ultrafast bootstrap (right). The unknown species is indicated by an asterisk.

The aligned plastid genome matrix contained 140 425 bp, of which 13 003 bp (9.26%) are variable and 3 790 bp (2.70%) are parsimony informative. The aligned nrITS matrix contained 724 bp, of which 324 bp (44.75%) are variable and 216 bp (29.83%) are parsimony informative. The best-fit model (TVM+F+R3) for plastid genome matrix and best-fit model (GTR+F+I+G4) for nrITS matrix were automatically chosen by IQTREE according to Bayesian Information Criterion. The phylogenetic analysis based on the plastid genome (Fig. 1) indicated that *Fordiophyton* is diphyletic and clustered with *Blastus*, *Bredia* and two species of *Phyllagathis* with weak support (SH-aLRT 76%, UFBoot 49%). Most species of *Fordiophyton* formed a well-resolved clade (Clade A), which includes the type of the genus, while only one species, *F.breviscapum* (Clade B), was placed sister to *Blastus*. Clade A can be further divided into two subclades (A1 and A2) with strong support (SH-aLRT 100%, UFBoot 100%). The unknown species is sister to *F.strictum* and three other *Fordiophyton* species in Subclade A2 with strong support (SH-aLRT 100%, UFBoot 100%). The phylogenetic analysis based on nrITS included all *Fordiophyton* species except *F.damingshanense* S.Y.Liu & X.Q.Ning and *F.degeneratum* (C.Chen) Y.F.Deng & T.L.Wu which supported that *Fordiophyton* is not monophyletic and the unknown species is clustered with four species of Yunnan and one species of Vietnam (Suppl. material 2: Fig. S1).

## ﻿Discussion

The phylogenetic analyses confirmed that *Fordiophyton* is polyphyletic and consists of two clades (Zhou et al. 2019a, b; Dai et al. 2020). In Clade A, the unknown species was clustered with four other sympatric *Fordiophyton* species from Yunnan in Subclade A2. The forked and curved anther base of the longer stamens seems to be a synapomorphy of this subclade. In contrast, the species in Subclade A1 usually have longer stamens with unforked anther bases and are distributed in Guangdong, with the exception of the widespread *F.faberi* (occurring in almost every province of southern China) which, unusually, has longer stamens with forked anther bases like species in Subclade A2.

Morphologically, the unknown species is more similar to *F.faberi* than any other *Fordiophyton* species in both vegetative and reproductive characters, such as the erect stems, long petioles, slightly oblique and membranous leaf blades, broadly ovate to suborbicular bracts, and non-auriculate base of the calyx lobes. But the phylogenetic analyses revealed the unexpected placement of the unknown species in a different subclade from *F.faberi* (as shown in Fig. 1). A detailed comparison of this unknown species, *F.faberi*, and *F.strictum* (as a representative of species in Subclade A2) is provided in Table 1.

**Table 1.** Morphological comparison of key features among *Fordiophytontereticaule*, *F.faberi* and *F.strictum*.

Characters	*F.tereticaule*	*F.faberi*	*F.strictum*
Stem	terete, with dense glandular trichomes and puberulous when young, becoming glabrescent	quadrangular, glabrous	quadrangular, glabrous, usually pilose or setose at nodes
Petioles	1–7 cm long, densely puberulous	1.5–7 cm long, glabrous or shortly setose near leaf blade	usually less than 0.8 cm long, glabrous
Leaf blades	elliptic, slightly oblique	broadly lanceolate, oblong, ovate, or rarely lanceolate, slightly oblique	broadly lanceolate, oblique
Bracts	broadly ovate to suborbicular	broadly ovate to suborbicular	cordate
Calyx lobes	triangular to ovate-triangular without auriculate base	triangular to lanceolate-triangular without auriculate base	broadly ovate-triangular with auriculate base
Longer stamens	anther base lengthened into a forked and curved spur	anther base lengthened into a forked spur	anther base lengthened into a forked and curved spur

In Clade B, *F.breviscapum* is sister to *Blastus* with strong support (SH-aLRT 100%, UFBoot 100%) but far apart from two species of *Kerriothyrsus* C.Hansen Clade (Fig. 1). However, the phylogenetic analysis based on nrITS indicated that it is sister to the *Kerriothyrsus* Clade with weak support (Dai et al. 2020; Suppl. material 2: Fig. S1). Further study is needed to resolve the generic assignment of this species.

## ﻿Taxonomic treatment

### ﻿ Fordiophyton tereticaule

Taxon classificationPlantaeMyrtalesMelastomataceae

S.Jin Zeng & N.H.Xia sp. nov.

A426BFE4-B812-53A5-AF46-545ABED45B93

urn:lsid:ipni.org:names:77298656-1

**Figure 2.**
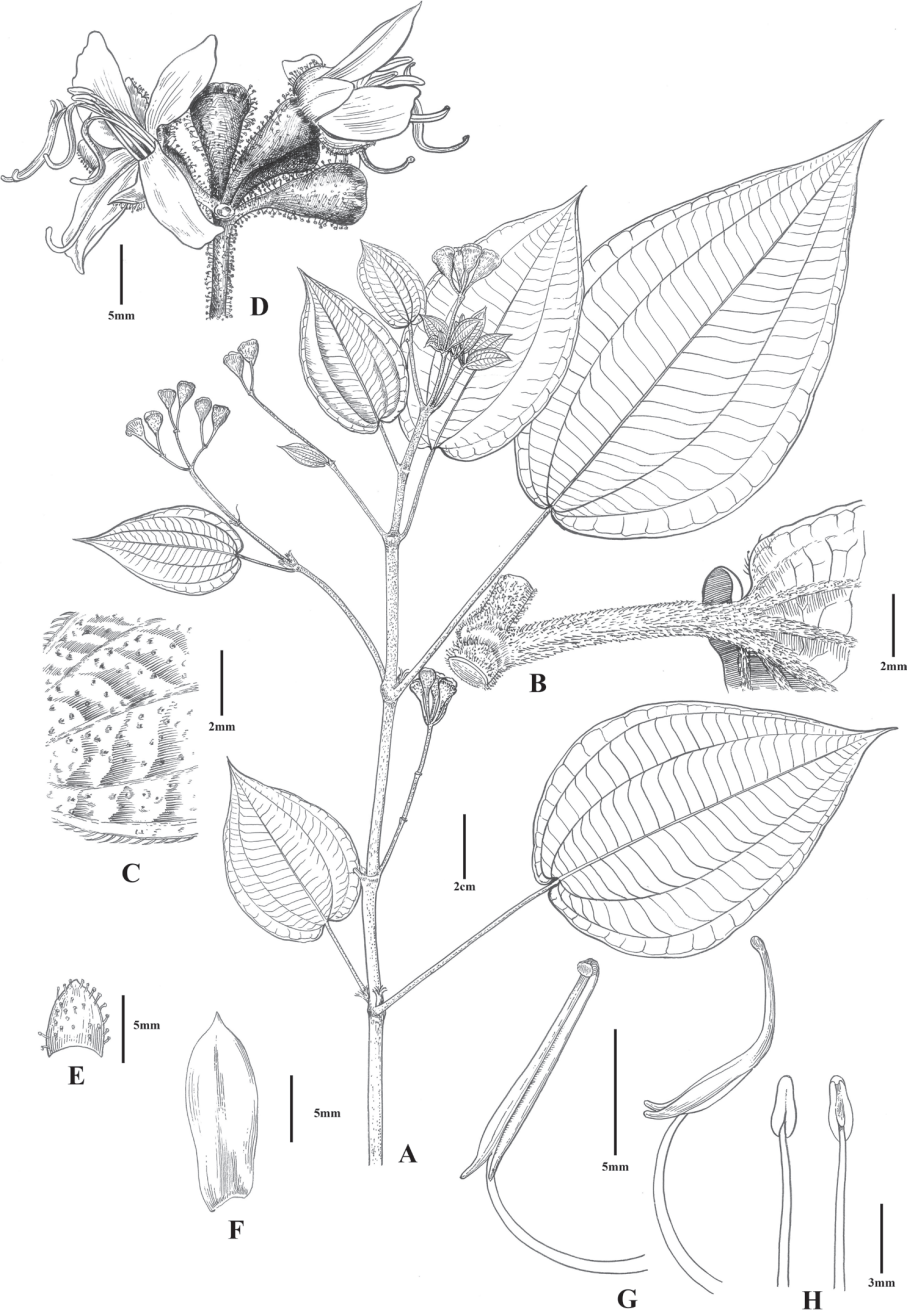
*Fordiophytontereticaule* S.Jin Zeng & N.H.Xia **A** fruiting branch **B** part of stem with one petiole **C** part of leaf blade, adaxial view **D** cymose inflorescence with opening flowers **E** calyx lobe **F** petal **G** longer stamen, front view (left) and side view (right) **H** shorter stamen, front view (left) and back view (right). Drawn by Ding-Han Cui.

**Figure 3.**
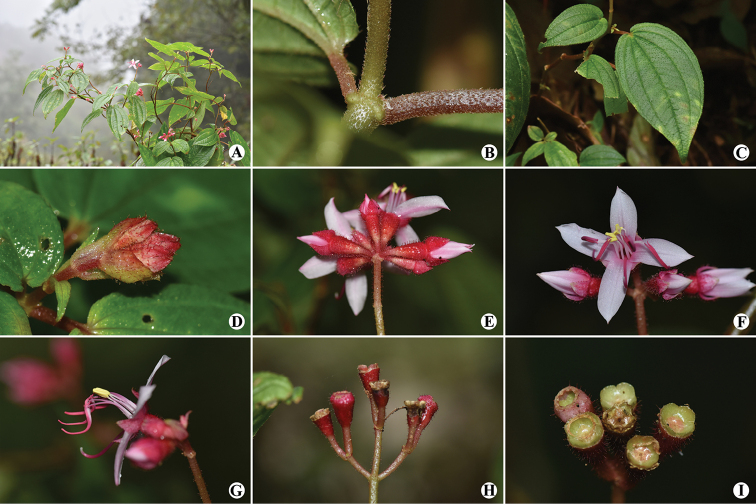
*Fordiophytontereticaule* S.Jin Zeng & N.H.Xia **A** flowering plants **B** part of stem with a pair of petioles **C** a pair of leaves **D** cymose inflorescence with flower buds **E** cymose inflorescence, oblique view **F** an opening flower, front view **G** an opening flower, side view **H** infructescence, side view **I** young fruits, top view.

Figs 2 , 3 Verbatim name: 圆茎异药花

#### Type.

China. Yunnan: Wenshan Zhuang and Miao Autonomous Prefecture, Malipo County, Laojunshan Provincial Nature Reserve, elev. 1517 m, 18 September 2020 (fl.), Si-Jin Zeng 4932 (holotype: IBSC!; isotypes: CANT!, KUN!, PE!).

#### Diagnosis.

Similar to *F.faberi* in having erect stems, membranous leaf blades, and oblong petals, but differs by the terete (vs. quadrangular) stems, densely puberulous (vs. green and glabrous or shortly setose near leaf blade base) petioles, and elliptic (vs. broadly lanceolate, oblong, ovate, or rarely lanceolate) leaf blades.

#### Description.

Herbs, 30–100 cm tall. Stems yellowish-green when young, turning red, terete, with dense glandular trichomes and puberulous when young, becoming glabrescent, inflated at the nodes. Leaves in unequal pairs in size; petiole reddish brown, terete, 1–7 cm long, densely puberulous; leaf blade elliptic, slightly oblique, 8–16 × 4–9 cm, membranous, base cordate, apex acuminate to caudate-acuminate, margin inconspicuously serrulate with each tooth bearing a terminal seta, adaxial surface yellowish-green, sparsely puberulous, abaxial surface yellowish-green, densely puberulous on veins; secondary veins 2–3 on each side of midvein, conspicuous; tertiary veins numerous, parallel, connecting with secondary veins. Inflorescences terminal, an umbel-like cyme, sometimes a thyrse, with 6–15 flowers; peduncle usually dull red, terete, 1.3–3.5 cm long, with dense red glandular trichomes; bracts yellowish-green to dull red, imbricate at base of pedicel, broadly ovate to suborbicular, with sparse red glandular trichomes, caducous. Pedicel dull red, terete, 3–5 mm long, with dense red glandular trichomes. Hypanthium scarlet, funnel-form, 6–8 × 3–4 mm, with dense red glandular trichomes. Calyx lobes dull red to scarlet, triangular to ovate-triangular, ca. 5 × 2–4 mm, with sparse red glandular trichomes, apex obtuse to acute, base not auriculate. Petals white to pinkish, oblong, ca. 1.1 × 0.5–0.6 cm, apex oblique with 1 glandular trichome at tip. Stamens 8, 4 longer antisepalous ones and 4 shorter antipetalous ones, arranged in 2 whorls. Antisepalous (longer) stamens 2.0–2.5 cm long; filaments 0.9–1.4 cm; anthers dull red, linear, ca. 1.1 cm long, curved, base lengthened into a forked, curved spur, connective base inflated. Antipetalous (shorter) stamens 0.9–1.2 cm long; filaments 0.6–0.9 cm; anthers yellow, ovoid, ca. 0.3 cm long, straight, connective base slightly inflated. Style 1.7–2.0 cm long, glabrous or with sparse glandular trichomes at base. Ovary half-inferior, ovoid, apex with a membranous crown. Capsule funnel-form, ca. 8 × 4 mm; placentation axillary, placentas shortly stalked; seeds more than 100, cuneate, less than 2 mm long.

#### Phenology.

Flowering in September-December, fruiting in October–next January.

#### Distribution and habitat.

*Fordiophytontereticaule* is only known from Malipo County, Yunnan, China. It grows in broad-leaved evergreen forests at elevations of 1260–1540 m.

#### Etymology.

The specific epithet refers to the terete stem.

#### Additional specimen examined.

China. Yunnan: the same locality as above, 1516 m, 28 November 2019, Si-Jin Zeng 898 (paratypes: IBSC!).

### ﻿Key to the species of *Fordiophyton*

**Table:** 

1	Internodes of stems indistinct, less than 2 mm	**2**
–	Internodes of stems distinct, more than 5 mm	**6**
2	Stem glabrous; petioles winged	**3**
–	Stem densely hirsute or setose; petioles unwinged	**4**
3	Petioles 8–18 cm long; leaf blades ovate or ovate-elliptic, 9–13 × 9–12 cm	***F.chenii***
–	Petioles 2–4 cm long; leaf blades elliptic, 4–9 × 2–4 cm	***F.zhuangiae***
4	Leaf blades with minute brown glands on both surfaces	***F.phamhoangii***
–	Leaf blades without minute brown glands on both surfaces	**5**
5	Adaxial surface of leaf blades glabrous; calyx lobes lanceolate, ca. 6 mm long	***F.huizhouense***
–	Adaxial surface of leaf blades tuberculate; calyx lobes triangular, 1–2 mm long	***F.peperomiifolium***
6	Leaves in a sub-basal rosette	**7**
–	Leaves cauline, not in a rosette	**9**
7	Petioles densely villous	***F.jinpingense***
–	Petioles glabrous or sparsely glandular-setose	**8**
8	Stem winged, glabrous at nodes; petioles glabrous; secondary veins 4 or 5 on each side of midvein	***F.cordifolium***
–	Stem unwinged, densely spiny at nodes; petioles sparsely glandular-setose; secondary veins 3 on each side of midvein	***F.brevicaule***
9	Stem creeping	***F.repens***
–	Stem erect or at least erect in the upper part	**10**
10	Leaves of a pair highly unequal (smaller one less than half of larger one’s size); petioles often less than 1 cm long; base of calyx lobes auriculate	***F.strictum***
–	Leaves of a pair not unequal or only slightly unequal; petioles often more than 2 cm long; base of calyx lobes not auriculate	**11**
11	Plants less than 25 cm tall; anthers of antipetalous stamens greatly reduced or sterile	**12**
–	Plants more than 30 cm tall; anthers of antipetalous stamens fertile, not greatly reduced	**13**
12	Stem winged; secondary veins 1 on each side of midvein	***F.breviscapum***
–	Stem unwinged; secondary veins 3 or 4 on each side of midvein	***F.degeneratum***
13	Stem terete; petioles densely puberulous	***F.tereticaule***
–	Stem quadrangular; petioles glabrous or shortly setose near leaf blade base	**14**
14	Abaxial surface of leaf blades furfuraceous; peduncle winged	***F.longipes***
–	Abaxial surface of leaf blades glabrous or minutely puberulous; peduncle unwinged	**15**
15	Inflorescence an umbel-like cyme or a thyrse; anther base of longer stamens not enlarged	***F.faberi***
–	Inflorescence a pleiochasium; anther base of longer stamens enlarged	***F.damingshanense***

## Supplementary Material

XML Treatment for Fordiophyton tereticaule

## Supplementary materials

Supplementary material 1 Table S1. Vouchers and Genbank accession numbers of the samples used in this study Data type docx file Explanation note Dashes (–) indicate missing data, and asterisks (*) denote the sequence obtained in this study. https://binary.pensoft.net/file/688532This dataset is made available under the Open Database License (http://opendatacommons.org/licenses/odbl/1.0/). The Open Database License (ODbL) is a license agreement intended to allow users to freely share, modify, and use this Dataset while maintaining this same freedom for others, provided that the original source and author(s) are credited.Si-Jin Zeng, Yi-Hua Tong, Nian-He Xia

Supplementary material 2 Figure S1. Phylogenetic relationships of Sonerileae based on nrITS Data type JPG file Explanation note The numbers near the nodes are the values of the SH-aLRT test (left) and ultrafast bootstrap (right). The unknown species is indicated by an asterisk. https://binary.pensoft.net/file/688533This dataset is made available under the Open Database License (http://opendatacommons.org/licenses/odbl/1.0/). The Open Database License (ODbL) is a license agreement intended to allow users to freely share, modify, and use this Dataset while maintaining this same freedom for others, provided that the original source and author(s) are credited.Si-Jin Zeng, Yi-Hua Tong, Nian-He Xia
