# Supplementary material for: ﻿Fordiophytontereticaule (Melastomataceae), a new species from China
Source: PhytoKeys. 2022 May 27;197:59–69. doi: 10.3897/phytokeys.197.82670 (PMC9849035; doi:10.3897/phytokeys.197.82670)
Supplement: Supplementary material 1 — Table S1. Vouchers and Genbank accession numbers of the samples used in this study [file phytokeys-197-059_article-82670__-s001.docx]

Table S1 Vouchers and Genbank accession numbers of the samples used in this study. Dashes (–) indicate missing data, and asterisks (*) denote the sequence obtained in this study.

| Taxon | Locality | Voucher | Plastid genome | | nrITS |
| --- | --- | --- | --- | --- | --- |
| *Allomorphia balansae* | Wuzhishan, Hainan, China | Liu 451 (SYS) | MK994792 | MG644470 | |
| *Allomorphia urophylla* | Jinping, Yunnan, China | Liu 718 (SYS) | MK994903 | MN031167 | |
| *Anerincleistus bracteatus* | Sarawak, Malaysia | Zhou et al. 698 (SYS) | MK994899 | MN031178 | |
| *Anerincleistus macrophyllus* | Sarawak, Malaysia | Zhou et al. 673 (SYS) | MK994887 | MN031184 | |
| *Anerincleistus setulosus* | Sabah, Malaysia | Zhou et al. 660 (SYS) | MK994881 | MN031185 | |
| *Blastus auriculatus* | Hekou, Nanxi, China | Liu 542 (SYS) | MK335944 | MG644463 | |
| *Blastus cochinchinensis* | Fengkai, Guandong, China | Liu 446 (SYS) | MK994909 | MG644465 | |
| *Blastus mollissimus* | Guiping, Guangxi, China | Liu 622 (SYS) | MK994863 | MN031195 | |
| *Bredia fordii* | Fengkai, Guangdong, China | Liu 444 (SYS) | MK994790 | MG644400 | |
| *Bredia hirsuta* | Okinawa, Japan | Liu 634 (SYS) | MK994872 | MN031213 | |
| *Bredia longiradiosa* | Longzhou, Guangxi, China | Liu 486 (SYS) | MK994807 | MN031235 | |
| *Cyphotheca montana* | Jinping, Yunnan, China | Liu 596 (SYS) | MK994852 | MG644447 | |
| *Fordiophyton brevicaule* | North District, Hongkong, China | Zeng et al. 43858 (CANT) | – | KM521841 | |
| *Fordiophyton breviscapum* | Ruyuan, Guangdong, China | Liu 441 (SYS) | MK994788 | MG644455 | |
| *Fordiophyton chenii* | Enping, Guangdong, China | Zeng Q008 (CANT) | – | KM521843 | |
| *Fordiophyton cordifolium* | Gaozhou, Guangdong, China | Liu 430 (SYS) | MK994784 | MN031160 | |
| *Fordiophyton faberi* | Emei, Sichuan, China | Liu 588 (SYS) | MK994846 | MN031161 | |
| *Fordiophyton huizhouense* | Huidong, Guangdong, China | Liu 433 (SYS) | MK994786 | MG644458 | |
| *Fordiophyton jinpingense* | Jinping, Yunnan, China | Liu 641 (SYS) | MK994875 | MN031162 | |
| *Fordiophyton longipes* | Pingbian, Yunnan, China | Liu 610 (SYS) | MK994858 | MN031163 | |
| *Fordiophyton peperomiifolium* | Qingyuan, Guangdong, China | Liu 432 (SYS) | MK994785 | MG644459 | |
| *Fordiophyton phamhoangii* | – | Liu 820 (SYS) | – | MT657307 | |
| *Fordiophyton repens* | Pingbian, Yunnan, China | Liu 513 (SYS) | MK994815 |  | |
| *Fordiophyton strictum* | Pingbian, Yunnan, China | Liu 514 (SYS) | MK994816 | MN031228 | |
| *Fordiophyton tereticaule* | Malipo, Yunnan, China | Zeng 898 (IBSC) | OM642120* | ON261402* | |
| *Fordiophyton zhuangiae* | Yangchuan, Guangdong, China | Liu 574 (SYS) | MK994839 | MG644462 | |
| *Kerriothyrsus tetrandrus* | – | Liu 794 (SYS) | – | MT657304 | |
| *Phyllagathis chongzuoensis* | Chongzuo, Guangxi, China | Zhang ZZ0824 (FJFC) | OL457073 | OL415178 | |
| *Phyllagathis elattandra* | Guiping, Guangxi, China | Liu 554 (SYS) | MK994830 | MG644431 | |
| *Phyllagathis erecta* | Malipo, Yunnan, China | Liu 507 (SYS) | MK994811 | MG644442 | |
| *Phyllagathis nanakorniana* | – | Sangrattanaprasert B203/14 (SYS) | – | MT657305 | |
| *Phyllagathis rotundifolia* | Kuala Lumpur, Malasysia | Zhou et al. M50 (SYS) | MK994912 | MG644436 | |
| *Phyllagathis subrotunda* | – | Liu 786 (SYS) | – | MT657308 | |
| *Phyllagathis tetrandra* | Xichou, Yunnan, China | Liu 519 (SYS) | MK994817 | MG644432 | |
| *Phyllagathis tuberosa* | – | Wai 2567 (SYS) | – | MT657306 | |
| *Plagiopetalum esquirolii* | Malipo, Yunnan, China | Liu 594 (SYS) | MK994851 | MN031202 | |
| *Plagiopetalum serratum* | Jinping, Yunnan, China | Liu 717 (SYS) | MK994902 | MN031170 | |
| *Sarcopyramis napalensis* | Shuifu, Yunnan, China | Liu 628 (SYS) | MK994868 | MN031207 | |
| *Sonerila cantonensis* | Lingshui, Hainan, China | Liu 449 (SYS) | MK994791 | MG644491 | |
| *Sporoxeia clavicalcarata* | Jinping, Yunnan, China | Liu 716 (SYS) | MK994901 | MN031176 | |
| *Sporoxeia latifolia* | Malipo, Yunnan, China | Liu 524 (SYS) | MK994820 | MN031201 | |
| *Sporoxeia petelotii* | Jinping, Yunnan, China | Liu 719 (SYS) | MK994904 | MN031173 | |
| *Styrophyton caudatum* | Malipo, Yunnan, China | Liu 615 (SYS) | MK994860 | MN031203 | |
